# 

**Published:** 1898-04-16

**Authors:** 


**Notices of Births, Deaths, and Marriages** are inserted in this column at a charge of 2s. 6d. for an announcement not exceeding four lines.

### NOTICE TO CORRESPONDENTS.

All MS., Letters, Books for Review, and other matters intended for the Editor should be addressed The Editor, "The Hospital," The Hospital Building, 28 & 29, Southampton Street, Strand, W.C.

The Editor cannot undertake to return rejected MS., even when accompanied by stamped directed envelope.

**NOTICE.—Vol. XXIII. of "The Hospital" (October, 1897, to March 1898), in handsome cloth binding, will shortly be on sale at the Office of this Journal, price 7s. 6d. Cases for binding the Numbers contained in this Volume, 1s. 6d. Index to Vol. XXIII., 2½d., post free.**

**The Monthly Part for April is now ready. Price 6d., by post 8d.**

### The Student of Medicine.

### APPOINTMENTS.

Miss L. Cunard-Cummins, L.R.C.S. and L.R.C.P.I., appointed Assistant Medical Officer of the Lincoln Lunatic Hospital. M. Coates, M.D., F.R.C.S.Eng., L.S.A., Retired Deputy Inspector-General, R.N., appointed Honorary Surgeon to the Philanthropic Society. A. W. Hare, M.B., B.Ch.Aberd., appointed Junior House Surgeon to the Borough Hospital, Birkenhead. E. Kennington, M.R.C.S., L.R.C.P., appointed Medical Officer for the No. 8 District of the Basingstoke Union. G. R. Turner, F.R.C.S.Eng., appointed Surgeon to St. George's Hospital. W. Washbourn, L.R.C.P.Lond., M.R.C.S.Eng., appointed Surgeon to the General Infirmary at Gloucester and the Gloucestershire Eye Institution. W. D. Wiggins, M.R.C.S., M.R.C.P.Lond., appointed Assistant Medical Officer to the Greenwich Union Infirmary. W. Wynne, M.B., appointed Medical Officer for the No. 2 District of the Rye Union.

